# Effects of Salicylic Acid on the Metabolism of Mitochondrial Reactive Oxygen Species in Plants

**DOI:** 10.3390/biom10020341

**Published:** 2020-02-21

**Authors:** Péter Poór

**Affiliations:** Department of Plant Biology, University of Szeged, Közép fasor 52, H-6726 Szeged, Hungary; poorpeti@bio.u-szeged.hu; Tel.: +36-62-544-307

**Keywords:** alternative oxidase, cytochrome *c*, glutathione, hexokinase, nitric oxide, programmed cell death, permeability transition pore, superoxide dismutase, voltage-dependent anion channel

## Abstract

Different abiotic and biotic stresses lead to the production and accumulation of reactive oxygen species (ROS) in various cell organelles such as in mitochondria, resulting in oxidative stress, inducing defense responses or programmed cell death (PCD) in plants. In response to oxidative stress, cells activate various cytoprotective responses, enhancing the antioxidant system, increasing the activity of alternative oxidase and degrading the oxidized proteins. Oxidative stress responses are orchestrated by several phytohormones such as salicylic acid (SA). The biomolecule SA is a key regulator in mitochondria-mediated defense signaling and PCD, but the mode of its action is not known in full detail. In this review, the current knowledge on the multifaceted role of SA in mitochondrial ROS metabolism is summarized to gain a better understanding of SA-regulated processes at the subcellular level in plant defense responses.

## 1. Introduction

Surviving negative effects of a wide variety of environmental fluctuations is a substantial part of plant life. Dynamic metabolic, genetic and morphologic changes of plants are necessary to acclimatize and/or adapt to harmful environmental conditions. These changes are controlled by the rapid transient or chronic production and the scavenging of reactive oxygen species (ROS) [1,2,3]. In these processes, several subcellular components have a distinguished role, such as mitochondria [4,5,6]. At the same time, the role of mitochondria can be investigated only together with other organelles (e.g., chloroplasts, nuclei, endoplasmic reticulum) in these processes, which can be mediated by various hormones, such as salicylic acid (SA) [7,8,9].

In this review, the current knowledge on the multifaceted role of SA on mitochondrial ROS metabolism is collected and summarized in plants to gain a better understanding of SA-regulated processes at the physiological, biochemical and molecular levels. This knowledge can add a new aspect to the understanding of mitochondrial oxidative stress signaling and its crosstalk with plant immune responses.

## 2. Basic Properties of Plant Mitochondria

Plant mitochondria are highly dynamic and pleomorphic subcellular organelles that are formed by at least six discrete compartments: the outer membrane, intermembrane space, inner boundary membrane, intracristal space, and matrix [10]. The shape (spherical, tubular, reticular), the size (0.2–1.5 μm) and the number of mitochondria (200–600) are highly variable depending on plant organs, cell type and physiological state [11,12,13,14]. Compared to animal mitochondrial genomes (ca. 16.5 kb), plant mitochondrial genomes range between 200–2000 kb encoding a small number of vital genes [15]. In addition, the mitochondrial matrix contains the pool of nicotinamide adenine dinucleotides (NAD, NADH), adenosine diphosphate (ADP), adenosine triphosphate (ATP), and the enzymes of the pyruvate dehydrogenase complex, tricarboxylic acid (TCA) cycle and glycine oxidative decarboxylation during photorespiration [10]. Moreover, plant mitochondria differ from their animal counterpart, with specific components of electron transport chain (ETC) and their functions such as photorespiration [16,17,18]. Furthermore, specific protein complexes of the mitochondrial outer membrane can also regulate immune response and communication with other organelles, such as chloroplasts or nucleus [4]. Based on these morphological and structural features, three essential functions of mitochondria can be distinguished: regulating bioenergetics, biosynthesis processes and signaling [4]. All of these functions are crucial to maintain cellular homeostasis, communication with other organelles (nucleus, chloroplasts, peroxisome, ER), and stress resistance. It has been shown that ROS generated by mitochondria are key signaling molecules in the regulation of these biological processes including hormone signaling [2,4,6,19,20,21].

## 3. Mitochondria-Associated ROS Metabolism and Non-ROS Molecules

In plant cells, the mitochondrial ETC is one of the major sites of ROS production. First of all, superoxide anion (O_2_^•–^) is generated under the normal respiratory and stressed conditions as well by mitochondrial ETC, due to the single electron leak to O_2_ in complex I [NADH–dehydrogenase], ubiquinone (UQ) and complex III [cytochrome *c* (cyt *c*)–oxidoreductase] [16,19,22,23]. The half-life of superoxide anion is 1–4 μs and the migration distance is in 30 nm [1].

Secondly, the produced O_2_^•–^ can be converted to H_2_O_2_ and O_2_ by a matrix-localized manganese-containing superoxide dismutase (MnSOD) enzyme. This enzyme is present both in the mitochondria and the peroxisome. MnSODs are either homodimers or homotetramers with one Mn (III) atom per subunit. Moreover, MnSODs are not inhibited by KCN, nor inactivated by H_2_O_2_ [24]. H_2_O_2_ is the most stable ROS (with a half-life of 1 ms) and it is very diffusible (more than 1 μm). It can be removed locally or exit from the matrix to the cytosol by aquaporins (AQP), where it can act as a signaling molecule (mediating tolerance e.g., by inducing antioxidant enzymes and hormone signaling) or as a toxic compound (inducing oxidative stress and cell death by damaging proteins, lipids, carbohydrates and nucleic acids) [1,5,6,19,25,26,27].

At the same time, not only can O_2_^•–^ be generated by mitochondrial ETC. Complex II and IV, as well as alternative oxidase (AOX) were also described as part of reductive pathways for nitric oxide (NO) synthesis, especially under low oxygen concentration [28,29]. NO can modulate protein structure or activity through S-nitrosylation of specific cysteine residues, through nitration of specific tyrosines or through binding to metal cofactors of the enzymes such as in the case of MnSOD [30]. NO is able to react with O_2_^•–^ generating peroxynitrite (ONOO^−^) and thus can regulate the redox status of the cell or participate in cell death induction. However, the basic ONOO^−^ concentration generated by mitochondria of tobacco plants is very low under unstressed condition based on the detection of confocal microscopy [31]. Delledonne and his co-workers found that ONOO^−^ in the concentration of 2 mM induced significant cell death in soybean cell suspension culture [32]. Peroxynitite is a strong oxidant, thus it may target and inhibit cysteine-containing thiols, such as tyrosine phosphatases [33]. Peroxynitrite can react with tyrosine and tryptophan residues yielding 3-nitrotyrosine and nitrotryptophane [34]. Tyrosine nitration can change the function of proteins, it may promote or inhibit the activity of enzymes, which is a developing field of current plant biology [35].

H_2_O_2_ can react with copper or iron ions in the Fenton reaction to generate hydroxyl radicals (OH^•^). OH^•^ is among the most highly reactive ROS (with a half-life of 1 ns and 1 nm mitigation distance), which can react with all biomolecules including nucleic acids, lipids and proteins [1,25,26]. The potential target of this process can be the [Fe-S]-containing bifunctional aconitase (ACO) protein, which plays a role in mediating oxidative stress and regulating cell death. This protein is localized both in the mitochondria and the cytosol. Under oxidative stress or by NO, the protein loses the [Fe-S] cluster and its enzymatic function, the reversible isomerization of citrate to isocitrate via cis-aconitate in the TCA cycle [36,37]. The abstraction of the hydrogen atom by OH· can cause the peroxidation of mitochondrial membrane polyunsaturated fatty acids (PUFA) leading to the formation of cytotoxic lipid aldehydes, alkenals and hydroxyalkenals (HAEs) such as 4-hydroxy-2-nonenal (HNE) and malondialdehyde (MDA), which are important markers of cell death [19,25].

Interestingly, diamine oxidase (DAO) activity has been identified in isolated mitochondria of *Helianthus tuberosu*s tubers [38], but the potential DAO activity has not been investigated in this cell compartment. DAO plays a role in polyamine catabolism showing a preference for putrescine (Put), and has a low affinity for spermidine (Spd) and spermine (Spm). It is known that increased activity of DAO releases H_2_O_2_ as a final product enhancing oxidative stress and plays role in the initiation of cell death [39].

**Figure 1:**
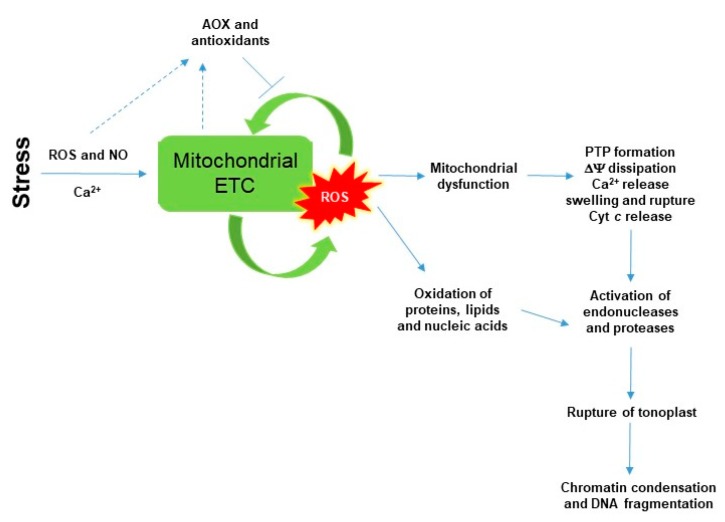
A schematic model for the role of mitochondria and mitochondrial reactive oxygen species (ROS) in programmed cell death (PCD) in plants. Briefly, stress-induced ROS and nitric oxide (NO) production, as well as Ca^2+^ inhibit the mitochondrial electron transport chain (ETC). This mitochondrial dysfunction increased mitochondrial ROS production in a self-amplifying manner leading the formation of permeability transition pore (PTP), dissipation of membrane potential (ΔΨ), loss of outer membrane integrity and release of cytochrome *c* (Cyt *c*) from the cytosol. Alternative components of plant mitochondrial ETC (e.g., alternative oxidase, AOX) and various mitochondrial antioxidants can attenuate mitochondrial ROS generation. However, several endonucleases and proteases are activated upon toxic ROS levels to degrade other cellular components such as vacuole membrane tonoplast and nuclei. Detailed description and references are in the text.

Peroxidation of mitochondrial membranes and high ROS production by ETC initiate the cyt *c* release from the mitochondrial inner membrane into the cytosol through the permeability transition pore (PTP; to be discussed in detail later) that contributes to the initiation of programmed cell death (PCD) in eukaryotes (Figure 1) [5]. Cyt *c* is a small heme-containing protein, which is a key component of mitochondrial ETC. It is associated loosely with the inner membrane of the mitochondria and transfers electrons between Complex III and IV. In animal cells, the release of cyt *c* to the cytoplasm drives the assembly of the apoptosome by binding to the apoptotic protease activating factor-1 (Apaf-1) and activating the caspase cascade through caspase 9 in the cytoplasm [40,41,42,43,44]. In plants, cyt *c* release activates cysteine proteases in the cytosol, moreover contributes to increase ROS content until lethal levels by blocking mitochondrial ETC (Figure 1). Structural changes in mitochondrial membranes are integral parts of this process. It was found in the non-plant cell that the phospholipid cardiolipin in the inner membrane of mitochondria undergoes peroxidation in the early step of apoptosis, which promotes the release of cyt *c* into the cytosol [45]. The significance of cardiolipin in plant mitochondrial architecture and physiology has been also confirmed by *Arabidopsis* knockout lines lacking *CARDIOLIPIN SYNTHASE 1* (*CLS1*), where transcript levels of antioxidant enzymes [e.g., catalase (CAT) and ascorbate peroxidase (APX)] showed significant differences compared to the wild-type plants [46,47]. However, cyt *c* release is associated not only to high ROS and lipid peroxidation but also to low ATP production, the collapses of mitochondrial transmembrane potential (ΔΨ) and the elevation of calcium levels [5,40,41,42,43,44].

AOX and uncoupling mitochondrial proteins (UCPs) are two mitochondrial energy-dissipating systems in plants [48,49]. The non-energy conserving terminal oxidase AOX plays a crucial role in the reduction of stress-induced ROS production in plants. AOX is a part of the mitochondrial ETC in plants, which directly couples the oxidation of UQ with the reduction of O_2_ to H_2_O bypassing complex III, cyt *c* and complex IV. At the same time, AOX reduces the energy (ATP) yield of respiration since it is not proton-pumping and as electrons flowing to AOX bypass the proton-pumping complex III and IV [50,51]. Therefore, AOX allows maintaining electron flow while simultaneously prevents the over-reduction of ETC [16,21]. It is well known that exogenous H_2_O_2_ treatment induced *AOX* expression in *Arabidopsis* [52] and that *AOX* expression can be attenuated by artificial ROS-scavengers (e.g., by *N*-acetylcysteine and flavone) in tobacco [53]. Moreover, it was showed that overexpression of AOX in plants can significantly alleviate mitochondrial-dependent PCD [54]. However, the knowledge of the diverse role of AOX in mitochondrial ROS metabolism could emerge in the future because its function could be specific under diverse environmental stimuli [23,50]. Like AOX, UCPs are also involved in the decrease of ROS production and membrane potential, while at the same time ROS are required for UCP activity [46,55,56,57].

The removal of toxic levels of H_2_O_2_ from mitochondria is mainly coordinated by the components of the glutathione-ascorbate cycle found both in mitochondria and chloroplasts [58]. H_2_O_2_ can be mostly degraded by CAT and APX or by several other enzymes (e.g., other peroxidases) to H_2_O [59]. Basically, the highest CAT activity was found in the peroxisomes. Additionally, mitochondrial and chloroplastic forms of CATs exist in maize and spinach [60]. APX oxidizes ascorbate (AsA) to monodehydroascorbate (MDHA) and dehydroascorbate (DHA). It has to be mentioned that AsA, similarly to glutathione (GSH), can be directly oxidized by ROS, however, these reactions show slower kinetics. In the regeneration part of the pathway, MDHA reductase (MDHAR), DHA reductase (DHAR) and glutathione reductase (GR) recycle the oxidized antioxidants to their reduced form. MDHAR and GR use NADPH, whereas DHAR requires GSH as a reducing equivalent [59]. AsA synthesis is associated with mitochondria, as it is synthesized in the intermembrane space and oxidized before being transported into the mitochondrial matrix (ca. 24 mM in the matrix), where it enters to the glutathione-ascorbate cycle to be reduced [16,23,60].

Similarly to AsA, GSH is also present in the mitochondrial matrix (ca. 6 mM), where it is transported by different transporters maintaining the constant pool of GSH [16,60]. Glutathione peroxidases (GPXs) can reduce H_2_O_2_ to H_2_O using GSH. It was shown that many of GPXs are localized to mitochondria in *Arabidopsis* [61]. Thus, GSH and GPX can also contribute to the defence against ROS damage. Moreover, the addition of a molecule of GSH causes S-Glutathionylation, the posttranslational modification of protein cysteine residues inactivating and protecting proteins in case of oxidative stress. Glutathionylation to deglutathionylation is manly catalyzed by glutaredoxin (GRX) [62]. Recently, it has been observed that only one GPX, GrxS15 is located in the mitochondria among the 33 GRXs in *Arabidopsis*, but GrxS15 is crucially important for lipoic acid-dependent enzymes in mitochondria, highlighting a putative role in the transfer of Fe-S clusters in this process [63].

Not only GRXs, thioredoxins (TRXs), which are ubiquitous small proteins, are also present in plant mitochondria (and nuclei). TRXs involve in the regulation of target proteins through the reduction of disulphide bonds maintaining protein dithiol/disulphide homeostasis [64,65,66]. At the same time, mitochondrial Trxo1 has been related to redox regulation of proteins, including AOX and to the detoxification of ROS via mitochondrial peroxiredoxin (PRX) IIF [67,68,69].

PRXs can decrease the level of mitochondrial H_2_O_2_ using reduced TRX or GSH as reductant sources, which in turn are reduced by thioredoxin reductase and GR [62]. At-PRXII F is one of six types II PRX identified in the genome of *Arabidopsis* and the only PRX that is targeted to the plant mitochondrion, which is essential for redox homeostasis by the decomposition of peroxides and by playing role in protecting the mitochondria during pathogen infection [62,70].

Plant glutathione transferases (GSTs) are also involved in the detoxification of a wide range of harmful compounds, including lipid peroxides, reactive aldehydes, and xenobiotics by the conjugation of GSH [71]. Plant GSTs consist of three superfamilies (cytosolic, mitochondrial, and microsomal) and can be further divided into distinct classes (e.g., tau, phi, theta, zeta, lambda), but the accurate function of mitochondrial GSTs remained unexplored [72,73].

The osmoprotectant proline (Pro) is also considered as a potent antioxidant and potential inhibitor of PCD. Pro has been proposed as an important molecule in redox signaling and inhibitor of lipid peroxidation, as well as OH· and superoxide scavenger [74]. Metabolism of Pro is associated with mitochondria. Catabolism occurs in this compartment catalyzed by Pro dehydrogenase (PDH) and P5C dehydrogenase (P5CDH) leading to the production of initial biosynthetic product glutamate (Glu) using FAD and NAD^+^ as electron acceptors [75]. When the activity of P5CDH is limited, the Δ1-pyrroline-5-carboxylate (P5C)-Pro cycle can transfer more electrons to the mitochondrial ETC and generate ROS in the mitochondria without producing Glu [76]. At the same time, it has been established that Pro participates in the protection of mitochondrial ETC Complex II [77].

## 4. SA and Its Effects on the Structure of Plant Mitochondria and ETC Compartments

ROS metabolism in the mitochondria and redox-mediated signaling cross-talk with plant hormones such as salicylic acid (SA). SA has been described to play an essential role in the regulation of plant defence signaling upon various abiotic and biotic stressors [78,79]. It is required for the establishment of both local and systemic acquired resistance (SAR) after a pathogen attack [80,81,82]. It is well known that there is a close correlation between ROS production and changes in the SA content [83]. Increase in the endogenous concentration of SA under various stress conditions induces the rapid accumulation of ROS, leading to oxidized proteins and cell death in the infected tissues [78]. However, SA can also induce stress tolerance that is highly dependent on the accumulation of superoxide radicals and H_2_O_2_, which are essential mediators of hypersensitive reaction (HR) and PCD induction in high concentration. Furthermore, SA-generated ROS could contribute to cellular redox homeostasis through the regulation of the expression and activity of antioxidant enzymes in lower levels [84]. At the same time, the source of ROS induced by SA could be originated from various cell compartments (e.g., chloroplasts, mitochondria, plasma membrane-localized NADPH oxidase, polyamine oxidase) [85,86,87]. Thus, the effects of different cell organelles to each other can further complicate unravelling the effect of SA. In addition, there are contrasting findings from different experiments in the case of SA-generated ROS. It has to be mentioned, that the action of SA is highly dependent, e.g., on its applied or internal concentrations, on the duration and the mode of the application, on the investigated plant species and organs as well as on environmental (e.g., light intensity) conditions [79,80]. Furthermore, the crosstalk between SA and other plant hormones (e.g., jasmonic acid and ethylene) can modify the defence reactions and PCD by regulating ROS metabolism [88]. In this section, the SA-generated mitochondrial ROS production, scavenging and signaling of mitochondrial ROS are summarized to understand the role of this important phytohormone in these processes.

SA can affect plant mitochondrial morphology and function in a dose- and time-dependent manner [7,9,87,89,90]. First of all, a rapid and significant change in mitochondrial morphology was observed in response to 0.5 mM SA in *Arabidopsis* protoplasts [8]. Authors found that tens of mitochondria arranged into clusters and the individual mitochondria became swollen within 40 min upon SA. After 1 h, a more irregular clumped or clustered morphology of mitochondria was observed in SA-treated protoplasts. Interestingly, the application of AsA before SA exposure reduced these alterations in mitochondrial morphology, which demonstrated that the aggregation of mitochondria is highly dependent on the production of mitochondrial ROS [8]. In intact tomato mesophyll cells, exogenous SA treatments caused swelling and disorganization of mitochondrial cristae as well as disintegration and vacuolization of mitochondria, which proved to be more serious at lethal (1 mM) SA concentration after 24 h [91]. Changes in mitochondrial morphology are related to physiological function and energy metabolism [92], which are important steps of plant PCD [43,44,45,46]. High SA concentration can cause the loss of mitochondrial integrity and cyt *c* release from mitochondria as well as ROS production and membrane lipid peroxidation, which take place before PCD execution [5,92]. At the same time, the long-term effect of sublethal concentration of SA on mitochondria biogenesis, number, structure and contact with other organelles have not been analyzed yet. Among others, investigation of prohibitins, which play role in the biogenesis and protection of mitochondria could be an interesting research field. Namely, earlier it was found that the suppression of prohibitin function resulted in a 10- to 20-fold higher ROS production and premature leaf senescence in *Nicotiana benthamiana*, and these plants were more susceptible to SA [93].

**Figure 2:**
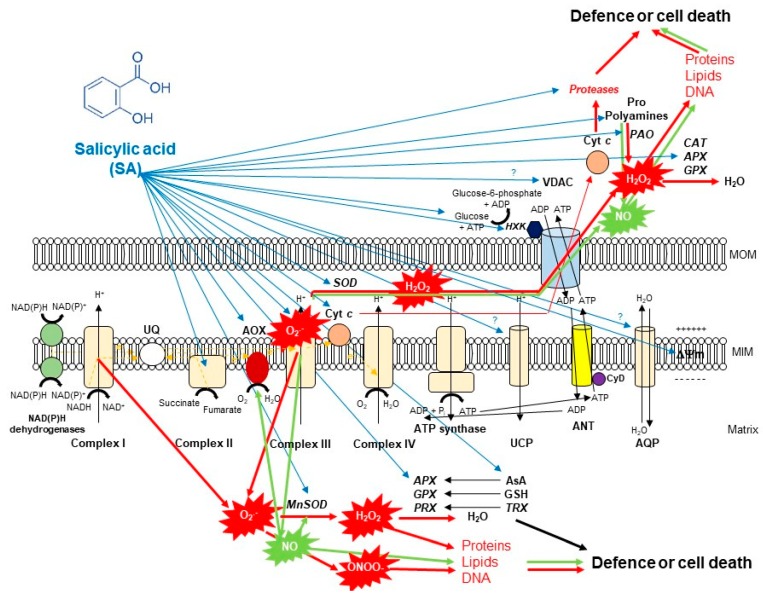
A schematic illustration of the effects of salicylic acid (SA) on the metabolism of reactive oxygen species (ROS) in plant mitochondria. The accumulation of endogenous SA leads to an increase in mitochondrial ROS and nitric oxide (NO) production. Mitochondrial complex III plays the dominant role in superoxide (O_2_^•−^) generation upon SA. O_2_^•−^ is able to react with NO generating peroxynitrite (ONOO^−^) and thus can regulate the redox status of the cell or initiate cell death. However, the produced O_2_^•−^ can be converted by superoxide dismutase (SOD) enzyme to H_2_O_2_. It was observed that exogenous SA treatments elevated the mitochondrial Mn-SOD activity and gene expression. The decomposition of H_2_O_2_ is mediated by several antioxidant enzymes (ascorbate peroxidase, APX; guiacol-peroxidases, POD; peroxiredoxin, PRX) and antioxidants (ascorbate, AsA; glutathione, GSH; thioredoxins, TRX) mediated by SA or H_2_O_2_ can exit from mitochondria playing role in cell signaling or oxidizing proteins, lipids and nucleic acids. Moreover, the SA-regulated alternative oxidase (AOX) plays a crucial role in the reduction of mitochondrial ROS and cell death mechanisms. The high concentration of SA not only induces toxic ROS production but also the release of cytochrome *c* (Cyt *c*) from the mitochondrial inner membrane through the permeability transition pore (PTP) formed by the voltage-dependent anion channel (VDAC) and the adenine nucleotide transporter (ANT) that contributes to the initiation of cell death. At the same time, mitochondrial hexokinases (HXKs) are important mediators of PTP regulation upon SA. However, the role of many component and molecule of mitochondria and cytosol (e.g., aquaporins, AQP; uncoupling mitochondrial proteins, UCP; polyamines such as spermine, Spm; proline, Pro) exposed to SA is not known in full details. Detailed description and references are in the text.

It has been also confirmed that SA by concentration- and time-dependent manner impact on mitochondria ETC by inhibiting the mitochondrial ETC and oxidative phosphorylation [7]. Firstly, it was found that 0.02–0.5 mM SA induced inhibition of both respiratory O_2_ uptake and ATP synthesis within minutes after SA incubation in tobacco cell suspension cultures. This effect of SA was reduced by the application of the antioxidant N-acetylcysteine, suggesting a possible role for ROS in the SA-mediated inhibition of mitochondrial functions [89]. Later, inhibition of O_2_ uptake of purified soybean cell mitochondria was also observed after 16 h-long-treatment with 1 mM SA [94]. Norman et al. [7] described firstly that SA at low concentrations (0.1–0.5 mM) acted as an uncoupler, whereas SA at higher concentrations (1–5 mM) strongly inhibited mitochondrial electron flow in tobacco cell suspension culture. Initially, they measured that SA blocks electron flow from the substrate dehydrogenases to the UQ pool [7]. Shugaev et al. [90] observed that by using stored taproots of sugar beet (*Beta vulgaris* L.) and etiolated seedling cotyledons of yellow lupine (*Lupinus luteus* L.), the uncoupling action on mitochondrial respiration and dissipation of mitochondrial membrane potential upon SA treatment was not only dependent on SA concentration but also on the duration of the treatment and on the sensitivity of mitochondria isolated from different plant tissues to the phytohormone. However, the direct effect of SA on ETC components remained unknown. Complex I and III of ETC are considered to the major sites of ROS production. Namely, over-reduction of ETC components and accumulation of mitochondrial ROS are well-characterized effects of Complex I inhibitor rotenone and Complex III inhibitor antimycin A (AA) [16]. Interestingly, it was found earlier that salicylate interacted with a Fe-S cluster of mitochondrial Complex I from rat liver which led to generation of ROS [95]. At the same time, rotenone did not induce significant ROS production in non-photosynthesizing cell suspension cultures of *Rubus fruticosus* suggesting that it did not affect the reverse electron transfer [96]. In contrast to Complex I, Complex III was described as the major source of ROS generation by inactivating the semiquinone radical during the Q cycle after 2.5 mM SA treatments in this cell suspension culture [96]. Later Nie et al. [8] demonstrated with fluorescence techniques that 0.5 SA might act directly on the complex III in plant mitochondrial ETC by inhibiting the respiratory activity and causing rapid oxidative burst within minutes in isolated *Arabidopsis* mitochondria (Figure 2). These observations underline the possible organ- and tissue-specific (photosynthetic or not photosynthetic) effects of SA on plant mitochondria. At the same time, the effects of SA on ETC complexes at lower concentration (<0.1 mM) remained unclear in isolated mitochondria of plants. Investigation of the role of SA at normal intercellular concentration is crucial because basal levels of total SA range between 1–10 μg FM^−1^ and it is elevated upon infection 10–100 fold higher depending on plant species [80,81]. Belt et al. [9] showed firstly that micromolar concentration of SA increased succinate dehydrogenase (SDH) activity but only when succinate-dependent electron transport was directed through the UQ binding site of SDH, elevating also the succinate:quinone reductase (SQR) activity. In addition, significant mitochondrial ROS production was observed after 7 mM SA treatment which was succinate-dependent using wild type *Arabidopsis* and *SDH1-1* (*dsr1*) and SDH assembly factor (*sdhaf2*) point mutant and knockdown plants [9]. These excellent articles provided the first quantitative and kinetic evidence for direct involvement of micromolar concentration of SA in an SDH-dependent signaling pathway in *Arabidopsis* that contributes to mitochondrial ROS production and leaves to SA-dependent transcriptional regulation.

## 5. SA-Induced Lethal Mitochondrial Oxidative Stress and Mitochondrial-Mediated Cell Death Induction

It is well known that SA can induce oxidative stress by concentration-dependent manner with the contribution of various cell organelles and processes (e.g., chloroplasts, mitochondria, plasma membrane-localized NADPH oxidase, polyamine oxidase) [85,86,91]. An interesting question is the network of various ROS sources and their interaction with each other. Which component of this network plays the initial first step? Is there any priming source of ROS to induce lethal oxidative stress? Is it dependent on the type of the stressed plant organs (root or leaves)? The excellent review article of Van Aken and Van Breusegem [5] showed a two-phase ROS burst model during PCD in plants. The first peak, which may originate from the apoplastic NADPH oxidase or possibly from energy-organelle sources such as mitochondria, resulting in activation of the mitochondrial PTP through ROS-activated Ca^2+^ influx. Based on this work, this results in membrane depolarization and ETC inhibition in mitochondria, causing a second large ROS burst that may also involve chloroplasts [5]. In addition, ROS levels are regulated by antioxidants and antioxidant enzymes upon e.g., SA during this period. Similar changes were observed after the treatment with exogenous SA in lethal 1 mM concentration in cell suspension culture [97] and in photosynthesizing tomato leaves but only one H_2_O_2_ peak was measured under dark condition [86]. Based on these observations, the source of the priming ROS burst and the role of energy-organelles in this process, as well as the interaction of various cellular compartments and ratio of pro- and antioxidants are crucial in the determination of cell fate, survive or death upon high SA levels.

The source of ROS production and the localization of its effects on various cell organelles are fundamental. SA-generated mitochondrial ROS can initiate and execute plant PCD. SA induced inhibition of mitochondrial ETC resulted in concentration-dependent ROS production in isolated mitochondria of *Arabidopsis* leaves. Significant ROS production was measured in the presence of respiration substrates malate plus glutamate (complex I substrate) or succinate (complex II substrate) in the presence of 0.5 mM SA [8]. In addition, ROS production and NO levels were also significant higher in isolated mitochondria from 1 mM SA-treated tomato leaves suggesting the direct effect of SA on mitochondrial ROS- and NO production [91].

Thus, SA in lethal concentration-generated high mitochondrial ROS causes mitochondrial dysfunction associated with morphology transition and depolarization of membrane potential. The mitochondrial membrane potential (ΔΨ) is a very sensitive indicator of the energy-coupling condition of mitochondria. It is generated by proton pumps (Complexes I, III and IV) which are essential components in the process of energy storage during oxidative phosphorylation. Together with the proton gradient (ΔpH) between the mitochondrial matrix and the intermembrane space, ΔΨ forms the transmembrane potential of hydrogen ions which drives ATP synthesis by F_O_F_1_-ATP synthase [98]. It has long been recognized that SA decreases mitochondrial ΔΨ (Figure 2). One of the earliest observation with pea mitochondria found that salicylate collapsed the transmembrane electrochemical potential and prevented both basal oxygen consumption and the activity of ATPase [99]. Significant decrease in mitochondrial ΔΨ was also reported after SA treatment. Moreover, the degree of Δψ dissipation was dependent on the applied phytohormone concentration and duration on isolated mitochondria from sugar beet taproots and lupine cotyledons [90,100], as well as in cell suspension cultures of *Rubus fruticosus* [96] and *Arabidopsis* leaves [101] or in protoplasts [8]. ROS-dependent disruption of mitochondrial morphology and collapse of ΔΨ are associated with the formation of PTP [102]. The integrity of the mitochondrial inner membrane is crucial for the optimal mitochondrial function. After the opening of PTP, the outer mitochondrial membrane becomes permeable to water and to large molecules (>1.5 kDa) and the influx of water causes swelling of mitochondria. Moreover, the ETC component cyt *c* is also released into the cytosol through the PTP that contributes to the initiation of PCD. However, the composition of the mitochondrial PTP is under strong debate [5]. Based on the model of Kusano et al. [103], the voltage-dependent anion channel (VDAC), the major protein of the outer mitochondrial membrane, the adenine nucleotide transporter (ANT) in the inner mitochondrial membrane and the matrix-localized cyclophilin-D are integral parts of PTP (Figure 2). Cyclosporin A and bongkrekic acid are pharmacological inhibitors of PTP opening, interacting with cyclophilin-D or ANT, respectively. PTP opening can be dependent on the accumulation of Ca^2+^ in the mitochondrial matrix and ADP/ATP ratio mediated by VDAC and ANT in plants and proapoptotic proteins (e.g., Bax) in animals [104]. Mitochondrial hexokinases (HXKs) are also key mediators of PTP regulation [105,106,107,108,109]. Mitochondrial HXKs can inhibit the PTP opening by binding to the VDAC at the cytosolic surface and the enzyme protein may act as a plug by blocking the channel [5]. Interestingly, SA can affect the activity and the expression of HXKs [91]. Recently, it was observed that both sublethal (0.1 mM) and lethal (1 mM) concentration of SA treatments decreased the activity and transcript levels of HXKs in leaves of tomato plants and the total mitochondrial HXK activity in the isolated mitochondrial fraction. Moreover, the potential effects of SA on VDAC were also confirmed by *AtVDAC2* transgenic *Arabidopsis* [110] but the interaction between VDAC and SA under PCD initiation remained unclear such as described earlier in case of methyl jasmonate [111].

At the same time, high ROS and NO production, as well as enhanced lipid peroxidation and cyt *c* release from mitochondria were also detected after 1 mM SA treatment [91]. Similar results of SA application were measured where SA triggered the release of cyt *c* from the mitochondria to the cytosol in *Arabidopsis* cell suspension culture [101] and in soybean seedlings [112]. Cyt *c* release is associated not only to high ROS and lipid peroxidation and to the collapse of ΔΨ but also to low ATP production and the elevation of Ca^2+^ levels [5,47]. It was earlier found that SA at concentrations of 0.05–0.5 mM induced rapid inhibition of both ATP synthesis and respiratory O_2_ uptake within minutes of SA incubation in tobacco cell suspension culture [89]. Moreover, the role of Ca^2+^ in SA-induced cell death was confirmed by application of Ca^2+^ chelator EGTA in tomato cell suspension culture [97] but changes in Ca^2+^ concentrations in mitochondria upon SA treatment remained undiscovered. However, changes of lipid composition in mitochondria upon SA has received less attention. These investigations could help to discover the multifaceted effects of SA on mitochondria. The study of Matos et al. [112] showed some interesting findings in the lipid composition and the respiratory properties in mitochondria of soybean hypocotyl. Authors observed that the phospholipid composition of mitochondria remained similar in control and SA-treated plants after 24 h, but a decrease in the relative amount of linolenic acid was measured in phosphatidylcholine, phosphatidylethanolamine and cardiolipin contents. Further investigation of the direct and indirect effects of SA on cardiolipin structure and biosynthesis would be necessary. Loss of mitochondrial integrity and cyt *c* release from mitochondria takes place before cell death execution [45,92]. This cyt *c* release can activate various cysteine proteases in the cytosol whose activities and the expression of its coding sequences were elevated by SA [97,101,113,114,115,116]. Cell death in plant organs is preceded by various cytological and biochemical features (Figure 1): specific morphological changes, rupture of vacuole membrane tonoplast, activation of proteases and nucleases, chromatin aggregation and DNA fragmentation [42,43,44,45,46,117], which hallmarks of PCD were also detected upon SA treatments [97,101].

## 6. SA-Mediated Defence against Toxic Mitochondrial Oxidative Stress: The Role of Alternative Oxidase (AOX)

The mitigation of toxic ROS levels generated by different cell compartments such as mitochondria is required at each stage of the life cycle. There are various strategies providing a key for high ROS which are mediated by phytohormones such as SA. It can be summarized that the primer source of ROS is originated from the inhibition of mitochondrial ETC. SA by concentration- and time-dependent manner can contribute to the elevated ROS production or scavenging and limiting of ROS in mitochondria of plants (Figure 2). First of all, the effects of SA on AOX is the most relevant and the most investigated topic. The role of AOX (together with UCP) is crucial when cyt *c* is released from mitochondria during the initiation of plant PCD because respiratory electron transport can continue under this circumstance [117,118,119,120]. In addition, AOX activity can help to decrease ROS production in mitochondria [121,122] and contribute to the NO production [28,29]. Moreover, changes in the expression of *AOX* genes have been proposed to represent an excellent ‘reporter gene’ to evaluate the mitochondrial dysfunction under stress conditions [104]. Effects of SA on AOX transcripts and protein abundance are highly-researched areas, particularly since the role of SA in thermogenic species *Sauromatum guttatum* has been documented [123]. Later, the induction of AOX by SA has been observed in various plant species such as in tobacco cell suspension culture after 12 h-long-treatment with 1 mM SA [124], in isolated mitochondria from tobacco leaves within 5 h after 1 mM SA treatment [125], in *Sauromatum guttatum* appendix after 0.01 mM SA treatment [126], in isolated soybean cell mitochondria after 16 h-long-treatment with 1 mM SA [94], in isolated tobacco cell mitochondria treated with 0.5 mM SA for 8 h [118], in *Orobanche* seeds exposed to 0.02 mM SA for 1–3 days [127], in tobacco calli after 8 h-long-treatment with 0.02 mM SA [128], and in purified mitochondria from the cotyledon of yellow lupine treated with 1 mM SA for 12 h [129]. It can be concluded that effects of SA on AOX protein level did not depend on the plant cell types (leaf, calli, cell suspension) and SA caused a rapid (within 24 h) changes in the AOX function.

Normann et al. [7] measured firstly concentration-dependent effects in case of SA treatments in tobacco cell suspension culture. 0.1 mM SA induced an increase in AOX protein levels which correlated with the increase in gene expression of *Aox1* after 4 h. In contrast to this observation, when 0.1 mM SA was applied, the measured increase in AOX was transient and disappeared as SA levels declined in the cells. At the same time, 0.01 mM SA also elevated the expression of other SA-responsive genes (e.g., *Pathogenesis-related 1*, *PR1*) but this effect of SA was dependent on active mitochondria [7]. Matos et al. [112] also measured that AOX capacity and protein contents increased after 24 h in mitochondria extracted from 1 mM SA-treated soybean seedlings. Interestingly, Authors observed that both *AOX1* and *AOX2b* transcripts accumulated in response to SA after 4 h but only *AOX2b* expression was significantly higher after 24 h [112]. These findings suggest also a concentration- and time-dependent effect of SA on the expression kinetics of AOX. This was further confirmed by the research of Cvetkovska and Vanlerberghe [130]. Surprisingly, the lethal concentration of SA at 3 mM failed to induce the expression of *Aox1a*, but 0.1 mM SA elevated *Aox1a* transcripts within 4 h and reduced cell death based on the detection of DNA laddering. In contrast to this observation, 0.5 mM SA promoted the accumulation of *Aox1a* transcripts after 4 h in tobacco cell suspension culture [130]. In tobacco leaf, an incompatible plant-bacteria interaction that produced high SA levels and HR was associated with low levels of AOX, whereas an incompatible interaction that produced only low SA levels with defence induction, but no HR, was associated with high levels of AOX [130]. Several studies about viruses revealed that SA-induced resistance is much simpler in Potato virus X (PVX) execution than SA-induced resistance to Tobacco mosaic virus (TMV) e.g., by mediating ROS [131]. It was observed that AOX-regulated defensive signaling is the predominant factor in controlling SA-initiated resistance during PVX infection [132].

Direct effects of SA on the transcriptional regulation of AOX could be an interesting research topic. Based on promoter analysis, the posttranscriptional mechanism of SA in the regulation of *AOX* coding sequences via H_2_O_2_ can take into account in the regulation of mitochondrial stress responses [52]. Transcript analysis in *Vigna unguiculata* leaves has also confirmed that treatment with 0.5 mM SA and 10 mM H_2_O_2_ induced a certain extent differently the expression of two AOX coding genes, *VuAox1* and *VuAox2b*. At the same time, both treatments caused a peak in *VuAox2b* expression after 6 h but the effects of SA were more prolonged on this gene [133]. In addition, NO has been also demonstrated to be an effective inducer of AOX gene expression [134,135], which could be also important in SA-mediated stress response. The opposite connection between SA and AOX has been also investigated, but this relationship needs further investigation. The study of Zhang et al. [136] hints that AOX level may (indirectly with ROS) influence SA level under biotic stress. In transgenic tobacco, which was silenced in the expression of AOX, the piercing-sucking insects (*Empoasca* spp.) caused more significant leaf damage. Also, HR-like cell death in response to bacterial infection (*Pseudomonas syringae* pv *tomato* DC3000) occurred more rapidly in the plants lacking AOX. Interestingly, in both cases, SA levels were significantly higher after hours in the challenged plants lacking AOX than in challenged wild-type plants [136].

## 7. SA-Mediated Defence against Toxic Mitochondrial Oxidative Stress: The Role of Enzymatic Antioxidants

Many studies have reported that SA regulates ROS levels by modulating antioxidants and the activity of key antioxidant enzymes including SOD, CAT and APX [79,80,137]. However, the timing and the role of the early activation/inactivation of various isoforms of these antioxidant enzymes under SA-induced ROS wave are less known, especially the interaction between the isoenzymes localized in different cell organelles such as in the mitochondria. Unfortunately, the analysis of these antioxidant enzymes and antioxidants from isolated mitochondria after SA treatment is mostly missing. There are only some studies which describe the effects of SA on mitochondrial isoenzymes investigated in organ level.

First of all, one of the most important is the analysis of the effects of SA on Mn-SOD (Figure 2). Mn-SOD is localized to mitochondria and peroxisomes and catalyzes the conversion of O_2_^•−^ generated by the inhibition of ETC to H_2_O_2_ [138]. It was measured that the activity of Mn-SOD declined in response to different *P. syringae* pathovars after 24 h similarly to the expression of *AOX1a* triggering HR by mitochondrial O_2_^•−^ in infected tobacco leaves [130]. In contrast to this observation, exogenous 1 mM SA treatment significantly elevated on gene-level the expression of chloroplastic *Cu/Zn-SOD* and mitochondrial *Mn-SOD* after 12 and 24 h in leaves of tomato plants [86].

Secondly, the fate of generated H_2_O_2_ is crucial (Figure 2). Most of H_2_O_2_ in plant cells is eliminated by CAT and APX [59]. It is well known that SA can bind directly to the CAT enzyme and inhibit the activity of certain isoenzymes in the peroxisome [139,140]. At the same time, SA induces the activity of APXs and guaiacol-peroxidases (POD), which also catalyze the decomposition of H_2_O_2_ to water but only in the cytosol [80,141]. However, there are also other enzymes, GPXs which protect against oxidative damage generated by ROS in plant mitochondria [61]. It was measured that 1 mM SA treatment caused high GPX activity and a significant increase in *GPX* expression within 24 h in soybean seedlings [112]. At the same time, only the results of Milla et al. [142] indicated that SA has an effect on mitochondria-related *GPXs*. There are only two GPXs displaying subcellular localization in mitochondria of *Arabidopsi*s. While *AtGPX3* showed only mitochondrial isoforms, *AtGPX6* may encode mitochondrial and cytoplasmic isoforms. Interestingly, a significant *AtGPX6* expression was found in ten-day-old *Arabidopsis* exposed to 1 mM SA for 12 h, whereas *AtGPX3* transcripts fell below the control level. However, another defence hormone treatment, jasmonic acid specifically promoted the expression levels of *AtGPX6* in this plant [142] suggesting the diverse signaling pathways mediated by these defence hormones. Thus, biochemical and specific physiological characterization of mitochondrial GPXs are needed in the future to understand the effects of SA.

## 8. SA-Mediated Defence against Toxic Mitochondrial Oxidative Stress: The Role of Non-Enzymatic Antioxidants

Not only enzymatic antioxidants but also changes in AsA and GSH levels showed strong time- and organ-specific (young leaf, old leaf, root) pattern after SA treatments in tomato plants [142]. GSH content was higher in young leaves while DHA was elevated in old leaves of tomato plants exposed to 0.1 mM SA [143]. Investigation of the effects of SA on mitochondria from sink and source organs could improve our knowledge about the oxidative stress response of plants mediated by several antioxidants and sugars such as the HXK substrate glucose (Figure 2). The subcellular distribution of GSH is also an important factor but not well-studied similarly to AsA. However, its relevance was confirmed in tobacco plants after TMV infection [144]. Highest levels of GSH were detected in mitochondria and the lowest ones in chloroplasts. Interestingly, mitochondria were the only organelles where TMV-inoculation resulted in a decrease of GSH levels when it compared to mock-inoculated plants [144]. GR mediates the reduction of oxidized glutathione (GSSG) to GSH with accompanying oxidation of NADPH to maintain the cellular redox state and counteract ROS toxicity [22]. It was found that GR-coding genes, *OsGR1* and *OsGR3* were dual-localized in the chloroplasts and mitochondria in rice, but the transcriptional regulation of these sequences was distinct. Based on gene expression analysis, *OsGR3* is highly induced by SA but not by methyl jasmonate in rice [145]. These findings suggest the organelle-specific response and regulation upon SA and underline the importance of the investigation of the interaction between various cell compartments under stress condition. Although SA-inducible GRX and TRX have been reported [146,147], relevant scientific data about the effects of SA treatment on mitochondrial GRX, TRX and PRX are not available providing new research topic in the future.

Besides the key antioxidants, there are other significant molecules in plant cells playing role in the direct or indirect detoxification of mitochondrial ROS such as polyamines and Pro (Figure 2). Although the role of DAO in mitochondria has not been investigated, there are some interesting results about the possible role of polyamines associated to plant mitochondria. It was found that Spm can activate both SA-induced protein kinase (SIPK) and wound-induced protein kinase (WIPK) but only upregulation of WIPK was observed within 24 h in tobacco leaves [148]. Additionally, 0.5 mM Spm promoted the expression of the *AOX*, which was disrupted by antioxidants and Ca^2+^ channel blockers suggesting the role of ROS and Ca^2+^ in mitochondrial dysfunction caused by Spm [146]. Surprising results about the effect of SA on Spm were published by Takács et al. [85]. Markedly different polyamine contents were measured under light and dark conditions after the 24-h-long treatment with 1 mM SA in tomato leaves. Put and Spm accumulation showed a significant increase in the dark and DAO activity was decreased, while Spm content and activity of DAO were enhanced in the light after one day in tomato plants. Similar tendencies were shown in the case of the expression pattern of various polyamine biosynthetic genes [85]. These results suggest the different function of SA in induced PA metabolism under diverse environmental conditions which may also depend on active chloroplast and mitochondria, respectively. It can be concluded that the interaction between SA and polyamines during mitochondria-mediated HR needs to be elucidated. Further studies are required to clarify the defensive role of polyamines associated to plant mitochondria.

Not only polyamines but also Pro levels can be highly dependent on SA and SA on Pro [138,149,150]. Pro catabolism takes place in mitochondria by the sequential oxidations of Pro to P5C by PDH and P5C to Glu by P5CDH [75]. PDH is located at the matrix side of the inner mitochondrial membrane. It was found that SA-mediated signaling plays a role in the induction of *AtPDH1* likely through non-expressor of PR1 (NPR1) and SA induction-deficient 2 (SID2) during the early stages of avirulent pathogen infection in *Arabidopsis* [151]. Interestingly, the silencing of PDH in *Arabidopsis* significantly reduced lethal lesion formation in the leaves following *Pseudomonas syringae* treatments and resulted in decreased resistance to avirulent bacterial inoculation, indicating that ROS produced by mitochondrial PDH markedly contributes to HR initiation [151]. However, additional studies could help to clarify more precisely the role of SA on mitochondria-associated Pro metabolism.

## 9. Conclusions and Future Perspectives

Phytohormones such as SA play an important role in various signaling pathways in cooperation with ROS coordinating plant responses under diverse environmental stimuli (Figure 2). At the same time, despite the increasing scientific results in the last two decades, the understanding of the mode of SA action on mitochondria and mitochondrial ROS metabolism is still far from being fully explained.

The direct and indirect effects of SA on the balance of ROS production and scavenging, the mitochondria-mediated retrograde signaling, as well as the mitochondria-mediated redox balance has to be further investigated. The effects of mitochondrial ROS on SA biosynthesis is also a key question. The ‘point-of-no-return’ threshold upon SA-generated lethal and self-amplifying ROS has to be further analyzed. Spatiotemporal analysis of interactions and relationships of ROS-producing cell organelles in this process could provide exciting opportunities for further explorations. Moreover, detection of interactions between mitochondria, the ratio of active and inactive (suicide) mitochondria, mitochondrial fusion and fission, and measurements upon SA can help to understand the role of SA in plant PCD. The interaction between mitochondrial ROS and NO in SA-induced PCD and stress tolerance could be also an important research field. Genomic approaches can provide insight into SA induced morphological changes in plant mitochondria. Tools of molecular biology can serve transgenic and mutant plants to describe the direct effects of SA on plant mitochondria. One of the key questions is the interaction between mitochondria and chloroplasts (ER and nucleus). A better understanding of the underlying mechanism upon SA in plant mitochondria in parallel with other organelles will helpfully yield insight into how to alleviate plant diseases and toxic environmental stimuli. Proteomic analysis can help to find specific (oxidated) proteins upon mitochondrial ROS induced by SA in cell signaling. Understanding the repair of mitochondrial ROS-mediated damage also needs much more research. Metabolomic profiling could help to understand the changes mediated by SA in a concentration-dependent manner. Effects of SA on mitochondrial transporters are neither known in full details. Ionomic analysis could provide further details to understand the SA-caused changes in mitochondria.

Understanding mitochondrial ROS metabolism represents an important future challenge. A deeper knowledge of the role of SA in this process can help to design novel strategies for oxidative stress- and plant protection management in agricultural research.
